# Realising a global One Health disease surveillance approach: insights from wastewater and beyond

**DOI:** 10.1038/s41467-024-49417-4

**Published:** 2024-06-22

**Authors:** Richard Hill, Grant D. Stentiford, David I. Walker, Craig Baker-Austin, Georgia Ward, Benjamin H. Maskrey, Ronny van Aerle, David Verner-Jeffreys, Edmund Peeler, David Bass

**Affiliations:** 1 Centre for Environment Fisheries and Aquaculture Science, Weymouth, Dorset UK; 2 Centre for Environment Fisheries and Aquaculture Science, Genomics Facility, Exeter, Devon UK; 3 WorldFish, Jalan Batu Maung, 11960 Bayan Lepas, Penang Malaysia

**Keywords:** Diseases, Environmental impact, Epidemiology

## Abstract

One Health is a recognition of the shared environment inhabited by humans, animals and plants, and the impact of their interactions on the health of all organisms. The COVID-19 pandemic highlighted the need for a framework of pathogen surveillance in a tractable One Health paradigm to allow timely detection and response to threats to human and animal health. We present case studies centered around the recent global approach to tackle antimicrobial resistance and the current interest in wastewater testing, with the concept of “one sample many analyses” to be further explored as the most appropriate means of initiating this endeavor.

## Abstract (web summary)

There is growing recognition of the need for a One Health approach for coordinated management of disease threats affecting humans, animals, and the environment. In this Perspective, the authors discuss a roadmap for a One Health disease surveillance system focusing on the example of wastewater surveillance.

## Why One Health and why now?

The COVID-19 pandemic has underscored the need to strengthen national surveillance systems to protect a globally connected world^1^. Pathogen surveillance is essential for early detection, risk assessment, preparedness, and the effective management of zoonotic diseases and pandemic threats and is a critical component of global efforts to safeguard public health and prevent the escalation of various infectious disease outbreaks. Pandemics have disrupted societies and impacted public health throughout human history^2^, and, yet, despite the recommendations of many expert groups, public health surveillance systems have not yet improved to the point where emerging infectious threats can be better anticipated and detected earlier^3^. Globally, existing pathogen surveillance systems face various challenges such as global health security gaps, underreporting and delays in reporting, both coupled to fragmented and incomplete surveillance data. A lack of coordination, and inadequate laboratory capacity within countries and across borders may also impede timely responses to various emerging pathogens. The limitations of existing surveillance systems have been exposed, particularly when dealing with novel pathogens or pathogens whose geographic range has extended into a new region^3^.

These deficiencies have underlined the critical importance of the One Health (OH) concept to tackle emerging disease threats. Factors including increased travel^4^, trade^5^, intensive farming practices^6^ and climate change^7^, may amplify risks associated with the emergence of various pathogens in humans. This is particularly true of zoonotic pathogens, and the potential for humans to come into direct contact with these organisms from various sources. The primary tenet to the OH concept is that it recognizes the health of people is closely connected to the health of animals and our shared wider environment. In practice, this means that experts from a range of sectors - notably human health, animal health, plant health and the environment – must synergistically build a detection and response infrastructure that emphasizes the sharing of information and the coordination of actions across multiple sectors^8^. This approach seems obvious: emerging disease agents such as zoonotic pathogens represent such a broad and complex threat that a single discipline, institution or country cannot respond alone^9^. A OH surveillance approach, combining existing disease surveillance with a broader range of human, animal and environmental sampling programmes to assess biotic and abiotic hazards, is likely to be the foundation of success in society’s ongoing and future pandemic preparedness^10^.

We present here a potential roadmap for developing and imbedding a OH disease surveillance system, utilizing an example of wastewater testing to detect and respond to emerging human health risks (e.g. zoonotic pathogens, pharmaceutical residues etc). Here we advocate better utilization of existing disease surveillance methodologies, such as wastewater surveillance, as a key means of establishing more effective OH surveillance programmes.

## Tripartite, quadripartite agreements and using AMR as a template

On 21^st^ March 2022 the four international agencies – the Food and Agriculture Organization of the United Nations (FAO), the World Organization for Animal Health (WOAH), the UN Environment Program (UNEP) and the World Health Organization (WHO), signed a landmark agreement to strengthen cooperation to sustainably balance and optimize the health of humans, animals, plants and the environment^11^. This quadripartite memorandum of understanding (MoU) provides for the first time a legal and structural framework for these four agencies to work together at the animal-human-environmental interface. This represents a critical step change in OH and global health in general. Alongside explicitly outlining the critical importance of OH, the MoU explicitly pinpoints antimicrobial resistance (AMR) as a key and specific area of work between the four agencies^11^. AMR is in fact an excellent cross-sectoral topic on which to base a wider OH vision for several key reasons: (1) it is a global issue of pressing public health relevance; (2) AMR impacts plants^12^, animals and humans, and has complex and multifaceted environmental and anthropogenic sources, sinks, interactions and dimensions and as such is a OH topic in its own right^13^, (3) perhaps most importantly, the tripartite agencies of FAO, WHO and WOAH have already successfully established a Global Action Plan on AMR, which was adopted in 2015 through decisions adopted by the World Health Assembly^11^. Indeed, in this landmark achievement, participant countries have agreed to put in place national action plans on AMR that are consistent with the Global Action Plan, and to implement relevant policies and plans to prevent, control and monitor AMR. Taken together, therefore we already have a global model of working from an internationally binding legal and methodological framework that can be used as a template for wider OH issues, and for which a successful approach can be co-opted^14^. More recently, the FAO, WHO and WOAH established the Tripartite Zoonoses Guide (TZG) to support countries in taking a multisectoral, OH approach to address zoonotic diseases. The TZG provides principles, best practices and options to assist countries in achieving sustainable and functional collaboration at the human-animal-environment interface. Utilizing this, as well as a broad framework similar to that which has already been successfully applied to AMR^14,15^—e.g. with a governance structure, a Global Action Plan agreed by the quadripartite, and associated national action plans^16^. Using AMR as an example, there are ongoing efforts to incorporate wider OH principles that have been published recently that serve as a useful blueprint for imbedding such approaches globally^16–20^^.^

## Wastewater based epidemiology: an exemplar for disease surveillance?

Human populations produce large amounts of wastewater, which contain a broad range of chemicals and biota depending on its source. Sewage is a particularly prevalent form of wastewater, and its production is an inevitable consequence of concentrated human population centres. When sewage enters natural watercourses, it can have immediate and direct negative impacts on environmental health though the introduction of pathogens^19^, toxic compounds^20^ and the development of eutrophic conditions^21^. Sewage and other wastewaters have also been shown to be a source of microplastics contaminating aquatic environments^22,23^, which may have downstream impacts on both environmental and human health^24^. Untreated sewage contains high levels of micro-organisms, the range and diversity of which vary from location to location and through time^25^. These pathogens may have a direct impact on environmental health, as well as downstream impacts on human health. Sewage, as a vector for transmission of pathogens into watercourses, has been studied for well over a century. Other examples of how faecally contaminated water may be ingested include (but are not limited to) direct use of contaminated water such as swimming^26,27^, or the consumption of food crops which has been irrigated with contaminated water^28^. Sewage and other wastewater are a source of a wide range of chemical pollutants which may be directly toxic^20^. However, studies also show the high prevalence of non-toxic, but biologically active compounds such as pharmaceuticals and their derivatives within wastewater^29^. Some of these compounds have been shown to have potential impacts on environmental health^30,31^ and may bioaccumulate in filter feeders such as bivalve molluscs, which pose risks to human and animal health^32^.

Why study wastewater in the context of disease surveillance? While wastewater is a known source of pollution to the environment, its use as a source of public health information is also becoming widely accepted. Faeces and urine contain high levels of pathogens and chemicals that are shed by those infected or taking medications. Detecting and quantifying these biological and chemical targets in sewage allows community wide disease surveillance to be carried out in a relative non-invasive way and with fewer samples required overall to see the same patterns. This practice, known commonly as Wastewater-Based Epidemiology (WBE) has been used to some extent for many decades since the discovery by Paul, Trask and Gard^33^, that poliovirus can be detected in urban wastewater. During the COVID-19 pandemic, the concept of WBE has been taken much further, with the implementation of many national and sub-national wastewater surveillance programmes globally^34^. With this increase in resource dedicated to wastewater surveillance, has come an interest for using WBE to monitor emergent pathogens as well as an interest in its potential to monitor antimicrobial resistance (AMR) and endemic human pathogens^35^. Many governments and international organisations now recognise WBE as a valuable approach to non-invasive detection and monitoring of disease outbreaks^36–38^. In the case of SARS-CoV-2, Morvan et al.^39^, showed the WBE methods could complement and strengthen traditional disease surveillance, improving public health outcomes. Additionally, Brunner et al.^40^ showed that WBE approaches were capable of tracking the spread of specific SARS-CoV-2 variants across England, a method that could potentially reduce the requirement for equivalent surveillance in clinical samples, thus providing a cheaper and less invasive alterative to traditional disease surveillance approaches. For non-notifiable pathogens such as norovirus, which also contribute a significant burden of human disease in communities, WBE approaches may prove to be a valuable source of prevalence data that would otherwise be difficult to generate from clinical reporting alone. Emerging human pathogens, with cryptic reservoirs and potential zoonotic routes of transmission are also now being targeted using WBE coupled to genomic surveillance methods^41^.

The use of analytical chemistry techniques with wastewater to quantify antimicrobial usages in communities has also been demonstrated by Holton et al.^42^, as have other pharmaceuticals^43^ and drugs of abuse^44^. This allows a greater understanding of human activities and disease burdens within communities that might otherwise go undetected. Wastewater’s status as both a pollutant and a source of vital health-related data make it an ideal sentinel for disease and an essential part of the OH toolkit. The development of techniques that allow simultaneous detection of chemicals, pathogens and other micro-organisms^45^, will facilitate a better understanding of how diseases are transmitted and in the environment. Analysis of multiple factors, such as chemical composition, biomarker detection and viral load, simultaneously from individual samples may also reduce the cost of sampling and paired data for different factors can give insight into co-morbidity. Perhaps more importantly, this understanding may help use to develop new strategies for disease mitigation and prevention.

## Operationalizing a more efficient environmental sampling approach

**Fig. 1:**
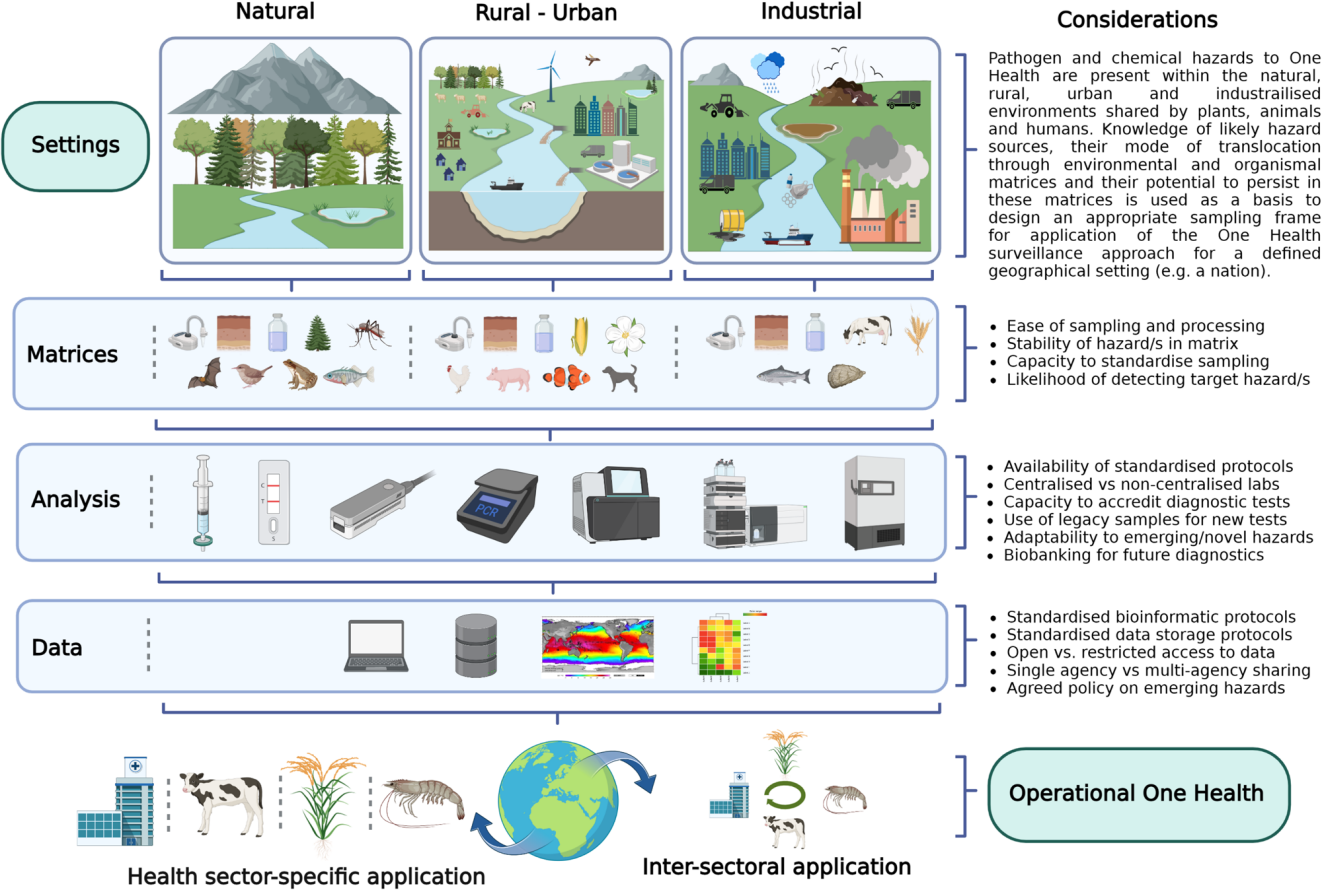
Schematic illustrating a possible work-flow for a transdisciplinary and operational One Health surveillance system, using three different environments (natural, rural/urban and industrialized habitats). The figure outlines a variety of key matrices that could be tested, as well as different laboratory analysis and data sources utilized. One sample many analyses (OSMA) is central to this approach. Created with BioRender.com released under a Creative Commons Attribution-NonCommercial-NoDerivs 4.0 International license.

**Fig. 2:**
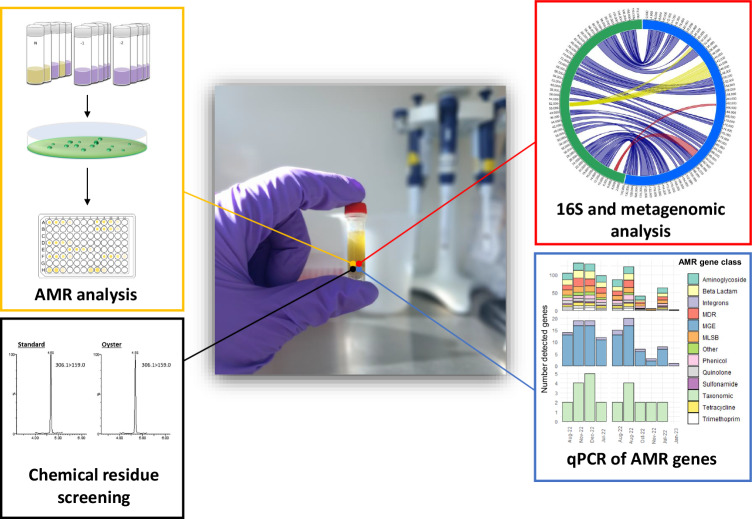
One sample = many analyses (OSMA). The broad array of tests that are available to look at emerging risks should be realized in an effective one health monitoring programme. An environmental sample, for instance, can be simultaneously analysed for the presence of AMR of bacteria and associated genes, and various molecular and chemical analyses to assess the use of water samples as sentinels of environmental health. Tube artwork reprinted from Walker, D., A., Younger, L. Stockley, and C. Baker-Austin. Escherichia coli testing and enumeration in live bivalve shellfish—present methods and future directions. Food Microbiology, Volume 73, August 2018, Pages 29–38., with permission from Elsevier.

What could a framework for surveillance look like? We propose that identifying key environmental conditions in each country/region and applying the principle of “one sample many analyses” (OSMA) would represent a more appropriate means of designing a OH surveillance programme. For example, begin by targeting sample collection according to the basic environmental setting in which they are acquired- natural, rural-urban, industrialized (Fig. 1). The second tenet to this approach, OSMA, is then applied in each setting (Fig. 2), such as that described for wastewater. Knowledge of the catchment size, likely health hazards and risks emerging from each setting would be crucial in developing sampling and testing regimes. Moreover, localized risk assessments and weighted risk analyses^46^ to identify possible “hotspots” of potential disease emergence could be an appropriate means of most effectively directing initial efforts. A similar approach has been implemented recently by the USAID Emerging Pandemic Threats Program (PREDICT) which focused efforts on strengthening zoonotic virus surveillance and laboratory capacity in “hotspot” areas^47^. Crucially, resources such as trained personnel, equipment, infrastructure and internet access are all vital to achieving a functioning laboratory system. This is notably challenging in lesser developed countries, therefore sharing of these resources across boundaries is to be encouraged as a means to achieving regional and global surveillance, and importantly, countries lacking these resources themselves can still receive surveillance data.

We have successfully applied similar approaches recently to study antimicrobial resistance in shellfish samples in the UK, where shellfish matrices from “impacted” and “reference” harvesting sites could be simultaneously analysed for AMR genes, AMR bacteria, pathogens and targeted chemical residues^48^. Such approaches offer the ability to use existing biological and chemical samples to assess ecosystem health and to detect and quantify specific emergent human health threats. One of the major expenses in any environmental or disease surveillance system is sample collection and transport prior to analysis, and so obtaining the maximum amount of data available for a single sample will greatly improve the efficiency of OH disease surveillance. The OSMA approach could be expanded across a broad range of sample types to encompass a variety of different environmental matrices as sentinels of ecosystem health, including air^49^, water^50^, soil^51^, aquatic and terrestrial animals as appropriate for each country and region. The application of chemical (e.g. mass spectrometry, MALDI-TOF, colorimetry) and biological (e.g. culture, PCR, genomics) analyses to samples can be used to obtain a wealth of data from individual samples in an efficient manner. We further propose the inclusion of samples that are already part of existing monitoring and disease surveillance efforts in-country which may already be tested for chemical and/or biological markers as part of statutory environmental, food safety or veterinary control measures. Centralized laboratories could develop and maintain standardized protocols for sampling, testing and data analysis that would be shared between and with partners and be freely accessible. The testing approaches used will vary between countries and OH laboratories, but we suggest that at a minimum they would include bacteriological and virological analyses, as well as metagenomic and chemical characterization of samples. For instance, rapid metagenomic approaches (as outlined here, Fig. 1) would include appropriate potential diagnostics, tracking pathogens and AMR genes, disease surveillance, and identification of key pathogens^52^. A parsimonious design would recognise that many aspects of sample treatment for molecular analyses are common to a range of assays. Therefore, assuming relevant sample matrices are available, a diverse range of biological entities could be assayed by core staff, using core techniques, on a minimal set of samples. The development of criteria for minimum acceptable data quality standards will also be essential to ensure the utility of the data to support OH policies within countries and globally. Resources such as trained personnel, electricity, clean water, reagents, internet access, equipment and maintenance of equipment are all vital. The appropriate long-term storage of samples would also be key: legacy/archived samples with appropriate metadata would be available for new tests and processing using novel technologies, and to retrospectively scrutinize emergent threats. This would require robust and standardized preservation and storage approaches for various OH samples.

Transforming these datasets into a comprehensible and actionable format within a reasonable timeframe presents a significant challenge. Historically, distinct datasets have been analyzed in isolated silos, each requiring specialized tools for fields such as genomics, remote sensing, ecology, climatology, oceanography, environmental science, and microbiology, among others (Fig. 1). Advancements in computational sciences, such as artificial intelligence and machine learning, are enabling the analysis of progressively larger volumes of data. By using a ‘big data’ framework, the integration of these data repositories has the potential to be of great value in this context, such as metagenomics-enabled disease surveillance methods which offer the opportunity to improve detection of both known and yet-to-emerge pathogens^17^. The development of a new generation of tools integrating multifaceted and composite data viewing approaches and shared between the OH laboratories could be a unifying vision for how data and outputs are ultimately shared and used. The success of the laboratories in each nation or region and more widely could be monitored against a defined set of key performance indicators underpinned by evidence, policy and legislation such as those proposed recently for OH in aquaculture settings^53^. A substantial challenge to the development of multisectoral One Health laboratories at the national level will be in specific requirements for national legislation and regulations that permit and support the sharing of diagnostic samples, testing procedures and results between laboratories/laboratory systems from the different sectors.

## Summary

Emerging diseases arise, for the most part, from wildlife but the majority of funds are spent on understanding and controlling diseases solely in humans^54^. It is clear that the OH concept should serve as the core of global efforts to prevent, respond and control emergent diseases, yet this concept is often overlooked within existing health and disease management structures. In this new era shaped by the COVID-19 pandemic, it is vital that policymakers, funders and the general public demand a comprehensive approach to public health mangement^10^. A key approach is to look across the entire gamut of existing approaches and with the example of WBE, identify tangible and practical approaches that work, and utilize an established surveillance framework. We believe that realizing this goal requires the formalization of a national and international network or laboratories dedicated to this critical work. This network should operate under a shared thematic and methodological framework focused on tackling OH-driven risks such as zoonotic pathogens.

## References

[1] Worsley-Tonks, K. E. L. et al. Strengthening global health security by improving disease surveillance in remote rural areas of low-income and middle-income countries. *Lancet Glob. Health* **10**, e579–e584 (2022). 10.1016/S2214-109X(22)00031-6 PMC8923676 35303467

[2] Morens, D. M., Daszak, P., Markel, H. & Taubenberger, J. K. Pandemic COVID-19 joins history’s pandemic legion *mBio* **11**, e00812–20 (2020). 10.1128/mBio.00812-20 PMC7267883 32471830

[3] Gardy, J. L. & Loman, N. J. Towards a genomics-informed, real-time, global pathogen surveillance system. *Nat. Rev. Genet.* **19**, 9–20 (2018). 10.1038/nrg.2017.88 PMC7097748 29129921

[4] Findlater, A. & Bogoch, I. I. Human mobility and the global spread of infectious diseases: a focus on air travel. *Trends in Parasitol.* **34**, 772–783 (2018). 10.1016/j.pt.2018.07.004 PMC7106444 30049602

[5] Jones, K. E. et al. Global trends in emerging infectious diseases. *Nature* **451**, 990–993 (2008). 18288193 10.1038/nature06536 PMC5960580

[6] Jones, B. A. et al. Zoonosis emergence linked to agricultural intensification and environmental change. *Proc. Natl Acad. Sci. USA* **110**, 8399–8404 (2013). 23671097 10.1073/pnas.1208059110 PMC3666729

[7] Mora, C. et al. Over half of known human pathogenic diseases can be aggravated by climate change. *Nat. Clim. Chang.* **12**, 869–875 (2022). 10.1038/s41558-022-01426-1 PMC9362357 35968032

[8] Ruckert, A., Zinszer, K., Zarowsky, C., Labonté, R. & Carabin, H. What role for one health in the COVID-19 pandemic? *Can. J. Public Health* **111**, 641–644 (2020). 10.17269/s41997-020-00409-z PMC7480204 32909226

[9] El Zowalaty, M. E. & Järhult, J. D. From SARS to COVID-19: a previously unknown SARS- related coronavirus (SARS-CoV-2) of pandemic potential infecting humans—call for a one health approach. *One Health* **9**, 100124 (2020). 10.1016/j.onehlt.2020.100124 PMC7075990 32195311

[10] Aarestrup F. M., B. M. K. M. Pandemics: One health preparedness for the next. *Lancet. Reg. Health Eur.* **9**, 100210 (2021). 10.1016/j.lanepe.2021.100210 PMC8495373 34642673

[11] World Health Organisastion. *Memorandum of Understanding between FAO and OIE and WHO and UNEP Regarding Cooperation to Combat Health Risks at the Animal-Human¬-Ecosystems Interface in the Context of the ‘One Health’ Approach and Including Antimicrobial Resistance*. 1–8 https://www.woah.org/app/uploads/2024/03/one-health-mou.pdf (2022).

[12] Miller, S. A., Ferreira, J. P. & Lejeune, J. T. Antimicrobial use and resistance in plant agriculture: a One Health perspective. *Agriculture* **12**, 289 (2022).

[13] McEwen, S. A. & Collignon, P. J. Antimicrobial resistance: a one health perspective. *Microbiol. Spectr.* **6**, 10.1128/microbiolspec.ARBA-0009-2017 (2018). 10.1128/microbiolspec.arba-0009-2017 PMC11633550 29600770

[14] World Health Organisation. *Global Action Plan on Antimicrobial Resistance*. 1–28 https://www.who.int/publications/i/item/9789241509763 (2015).

[15] World Health Organisation. *WHO Implementation Handbook for National Action Plans on Antimicrobial Resistance: Guidance for the Human Health Sector*. 1–67 https://www.who.int/publications/i/item/9789240041981 (2022).

[16] FAO UNEP WHO & WOAH. *One Health Joint Plan of Action, 2022–2026. One Health Joint Plan of Action, 2022–2026 (FAO; UNEP; WHO; World Organisation for Animal Health (WOAH) (founded as OIE)*. 1–70 https://iris.who.int/handle/10665/363518 (2022).

[17] OIE. *Joint Risk Assessment Operational Tool (JRA OT) An Operational Tool of the Tripartite Zoonoses Guide Taking a Multisectoral,**One Health Approach: A Tripartite Guide to Addressing Zoonotic Diseases in Countries*. 1–166 https://www.who.int/initiatives/tripartite-zoonosis-guide/joint-risk-assessment-operational-tool (2020).

[18] FAO WHO & WOAH. *Surveillance and Information Sharing Operational Tool An Operational Tool of the Tripartite Zoonoses Guide*. 1–70 https://www.who.int/initiatives/tripartite-zoonosis-guide/surveillance-and-information-sharing-operational-tool (2022).

[19] Xie, Y. et al. Insight into impact of sewage discharge on microbial dynamics and pathogenicity in river ecosystem. *Sci. Rep.* **12**, 6894 (2022). 35477966 10.1038/s41598-022-09579-x PMC9044725

[20] Costello, M. J. & Read, P. Toxicity of sewage sludge to marine organisms: a review. *Mar. Environ. Res* **37**, 23–46 (1994). 10.1016/0141-1136(94)90061-2

[21] Jarvie, H. P., Neal, C. & Withers, P. J. A. Sewage-effluent phosphorus: a greater risk to river eutrophication than agricultural phosphorus? *Sci. Total Environ.* **360**, 246–253 (2006). 16226299 10.1016/j.scitotenv.2005.08.038

[22] Kang, H.-J. et al. Occurrence of microplastics in municipal sewage treatment plants: a review. *Environ. Anal. Health Toxicol.* **33**, e2018013–0 (2018). 10.5620/eht.e2018013 PMC6182249 30286589

[23] Rolsky, C., Kelkar, V., Driver, E. & Halden, R. U. Municipal sewage sludge as a source of microplastics in the environment. *Curr. Opin. Environ. Sci. Health* **14**, 16–22 (2020). 10.1016/j.coesh.2019.12.001

[24] Walker, D. I. et al. A critical review of microbiological colonisation of Nano- and Microplastics (NMP) and Their Significance to the food chain. https://www.food.gov.uk/research/foodborne-disease/a-critical-review-of-microbiological-colonisation-of-nano-and-microplastics-nmps-and-their-significance-to-the-food-chain (2022).

[25] Shanks, C. et al. Comparison of the microbial community structures of untreated wastewaters from different geographic locales. *Appl. Environ. Microbiol* **79**, 2906–2913 (2013). 23435885 10.1128/AEM.03448-12 PMC3623150

[26] Rafiee, M., Hosseini, S. A., Gholami-Borujeni, F., Hesami Arani, M. & Niknejad, H. Health risk assessment of swimming beaches microbial contamination: a case study—Mahmoudabad, Iran. *Int. J. Environ. Health Res.* **34**, 355–366 (2022). 10.1080/09603123.2022.2149711 36446029

[27] Adriana, G.-F. et al. Risk of gastroenteritis from swimming at a wastewater-impacted tropical beach varies across localized scales. *Appl. Environ. Microbiol.* **89**, e01033–22 (2023). 10.1128/aem.01033-22 PMC10057883 36847564

[28] Berger, C. N. et al. Fresh fruit and vegetables as vehicles for the transmission of human pathogens. *Environ. Microbiol.* **12**, 2385–2397 (2010). 20636374 10.1111/j.1462-2920.2010.02297.x

[29] Samal, K., Mahapatra, S. & Hibzur Ali, M. Pharmaceutical wastewater as emerging contaminants (EC): treatment technologies, impact on environment and human health. *Energy Nexus* **6**, 100076 (2022). 10.1016/j.nexus.2022.100076

[30] Bisognin, R. P. et al. Potential environmental toxicity of sewage effluent with pharmaceuticals. *Ecotoxicology* **29**, 1315–1326 (2020). 32797393 10.1007/s10646-020-02264-7

[31] Gorovits, R., Sobol, I., Akama, K., Chefetz, B. & Czosnek, H. Pharmaceuticals in treated wastewater induce a stress response in tomato plants. *Sci. Rep.* **10**, 1856 (2020). 32024917 10.1038/s41598-020-58776-z PMC7002738

[32] Maskrey, B. H., Dean, K., Morrell, N. & Turner, A. D. A simple and rapid ultra–high-performance liquid chromatography–tandem mass spectrometry method for the quantitation of pharmaceuticals and related compounds in mussels and oysters. *Environ. Toxicol. Chem.* **40**, 3263–3274 (2021). 33760266 10.1002/etc.5046

[33] Paul, J. R., Trask, J. D. & Gard, S. Poliomyletic virus in urban sewage. *J. Exp. Med.* **71**, 765–777 (1940). 19870997 10.1084/jem.71.6.765 PMC2135110

[34] COVIDPoops19. *Summary of Global SARS-CoV-2 Wastewater Monitoring Efforts By UC Merced Researchers*. https://www.arcgis.com/apps/dashboards/c778145ea5bb4daeb58d31afee389082 (2023).

[35] Diamond, M. B. et al. Wastewater surveillance of pathogens can inform public health responses. *Nat. Med.* **28**, 1992–1995 (2022). 10.1038/s41591-022-01940-x 36076085

[36] European Union. Commission recommendation (EU) 2021/472 of 17 March 2021 on a common approach to establish a systematic surveillance of SARS-CoV-2 and its variants in wastewaters in the EU. *EUR-Lex* **98**, 3–8 (2021).

[37] HM UK Government. *Policy Paper—UK Biological Security Strategy.* https://www.gov.uk/government/publications/uk-biological-security-strategy/uk-biological-security-strategy-html (2023).

[38] G7 Health Ministers. *G7 Health Ministers’ Communiqué 20th May 2022.* https://www.g7germany.de/resource/blob/974430/2042058/5651daa321517b089cdccfaffd1e37a1/2022-05-20-g7-health-ministers-communique-data.pdf (2022).

[39] Morvan, M. et al. An analysis of 45 large-scale wastewater sites in England to estimate SARS-CoV-2 community prevalence. *Nat. Commun.* **13**, 4313 (2022). 35879277 10.1038/s41467-022-31753-y PMC9312315

[40] Brunner, F. S. et al. Utility of wastewater genomic surveillance compared to clinical surveillance to track the spread of the SARS-CoV-2 Omicron variant across England. *Water Res.* **247**, 120804 (2023). 37925861 10.1016/j.watres.2023.120804

[41] Treagus, S. et al. Metabarcoding of hepatitis E virus genotype 3 and norovirus GII from wastewater samples in England using nanopore sequencing. *Food Environ. Virol.* **15**, 292–306 (2023). 37910379 10.1007/s12560-023-09569-w PMC7615314

[42] Holton, E. & Kasprzyk-Hordern, B. Multiresidue antibiotic-metabolite quantification method using ultra-performance liquid chromatography coupled with tandem mass spectrometry for environmental and public exposure estimation. *Anal. Bioanal. Chem.* **413**, 5901–5920 (2021). 34498102 10.1007/s00216-021-03573-4 PMC8425450

[43] Ceolotto, N. et al. A new wastewater-based epidemiology workflow to estimate community wide non-communicable disease prevalence using pharmaceutical proxy data. *J. Hazard Mater.* **461**, 132645 (2024). 37793253 10.1016/j.jhazmat.2023.132645

[44] Baker, D. R. & Kasprzyk-Hordern, B. Multi-residue analysis of drugs of abuse in wastewater and surface water by solid-phase extraction and liquid chromatography–positive electrospray ionisation tandem mass spectrometry. *J. Chromatogr. A* **1218**, 1620–1631 (2011). 21334631 10.1016/j.chroma.2011.01.060

[45] Kasprzyk-Hordern, B. et al. Wastewater-based epidemiology for comprehensive community health diagnostics in a national surveillance study: mining biochemical markers in wastewater. *J. Hazard Mater.* **450**, 130989 (2023). 36848844 10.1016/j.jhazmat.2023.130989

[46] WHO. *A Tripartite Guide to Addressing Zoonotic Diseases in Countries Taking a Multisectoral.* 1–16 https://www.who.int/publications/i/item/9789241514934 (2019).

[47] Kelly, T. R. et al. Implementing One Health approaches to confront emerging and re-emerging zoonotic disease threats: lessons from PREDICT. *One Health Outlook* **2**, 1 (2020). 33824944 10.1186/s42522-019-0007-9 PMC7149069

[48] Environment Agency & Cefas. *Shellfish as Bioindicator for Coastal Antimicrobial Resistance - Summary*. 1–51 https://assets.publishing.service.gov.uk/media/6540c73546532b000d67f5bb/Shellfish_as_bioindicators_for_coastal_antimicrobial_resistance_-_report.pdf (2023).

[49] Littlefair, J. E. et al. Air-quality networks collect environmental DNA with the potential to measure biodiversity at continental scales. *Curr. Biol.* **33**, R426–R428 (2023). 37279659 10.1016/j.cub.2023.04.036

[50] O’Brien, E. & Xagoraraki, I. A water-focused one-health approach for early detection and prevention of viral outbreaks. *One Health* **7**, 100094 (2019). 10.1016/j.onehlt.2019.100094 PMC6501061 31080867

[51] Montgomery, D. R., Rabinowitz, P., Sipos, Y. & Wheat, E. E. Soil health: a common focus for one health and planetary health interventions. *One Health* **18**, 100673 (2024). 10.1016/j.onehlt.2023.100673 PMC10820383 38283832

[52] Ko, K. K. K., Chng, K. R. & Nagarajan, N. Metagenomics-enabled microbial surveillance. *Nat. Microbiol.* **7**, 486–496 (2022). 35365786 10.1038/s41564-022-01089-w

[53] Stentiford, G. D. et al. Sustainable aquaculture through the one health lens. *Nat. Food* **1**, 468–474 (2020). 37128071 10.1038/s43016-020-0127-5

[54] Mackenzie, J. S., McKinnon, M. & Jeggo, M. In *Confronting Emerging Zoonoses: The One Health Paradigm* (eds Yamada, A. et al.) 163–189 (Springer Japan, 2014).

