# Author Correction: Realising a global One Health disease surveillance approach: insights from wastewater and beyond

**DOI:** 10.1038/s41467-024-50682-6

**Published:** 2024-07-30

**Authors:** Richard Hill, Grant D. Stentiford, David I. Walker, Craig Baker-Austin, Georgia Ward, Benjamin H. Maskrey, Ronny van Aerle, David Verner-Jeffreys, Edmund Peeler, David Bass

**Affiliations:** 1 Centre for Environment Fisheries and Aquaculture Science, Weymouth, Dorset UK; 2 Centre for Environment Fisheries and Aquaculture Science, Genomics Facility, Exeter, Devon UK; 3 WorldFish, Jalan Batu Maung, 11960 Bayan Lepas, Penang Malaysia

**Keywords:** Diseases, Environmental impact, Epidemiology

Correction to: *Nature Communications* 10.1038/s41467-024-49417-4, published online 22 June 2024

In this article the author’s name Grant D. Stentiford was incorrectly written as Grant G. Stentiford. The original article has been updated.

